# Identification of new biomarkers for human papillary thyroid carcinoma employing NanoString analysis

**DOI:** 10.18632/oncotarget.3452

**Published:** 2015-03-26

**Authors:** Zhanna Chitikova, Marc Pusztaszeri, Anne-Marie Makhlouf, Margaret Berczy, Celine Delucinge-Vivier, Frederic Triponez, Patrick Meyer, Jacques Philippe, Charna Dibner

**Affiliations:** ^1^ Department of Medical Specialties, Faculty of Medicine, University of Geneva, Geneva, Switzerland; ^2^ Division of Clinical Pathology, University Hospital of Geneva, Switzerland; ^3^ iGE3Genomics Platform, University of Geneva, Switzerland; ^4^ Department of Thoracic and Endocrine Surgery, University Hospital of Geneva, Geneva, Switzerland; ^5^ Division of Endocrinology, Diabetes, Hypertension and Nutrition, University Hospital of Geneva, Geneva, Switzerland

**Keywords:** papillary thyroid carcinoma, NanoString analysis, FFPE, biomarkers, circadian clock

## Abstract

We previously reported an upregulation of the clock transcript *BMAL1,* correlating with *TIMP1* expression in fresh-frozen samples from papillary thyroid carcinoma (PTC). Since frozen postoperative biopsy samples are difficult to obtain, we aimed to validate the application of high-precision NanoString analysis for formalin-fixed paraffin-embedded (FFPE) thyroid nodule samples and to screen for potential biomarkers associated with PTC. No significant differences were detected between fresh-frozen and FFPE samples. NanoString analysis of 51 transcripts in 17 PTC and 17 benign nodule samples obtained from different donors and in 24 pairs of benign and PTC nodules, obtained from the same donor (multinodular goiters), confirmed significant alterations in the levels of *BMAL1, c-MET*, *c-KIT*, *TIMP1,* and other transcripts. Moreover, we identified for the first time alterations in *CHEK1* and *BCL2* levels in PTC. A predictive score was established for each sample, based on the combined expression levels of *BMAL1*, *CHEK1*, *c-MET*, *c-KIT* and *TIMP1*. In combination with *BRAF* mutation analysis, this predictive score closely correlated with the clinicopathological characteristics of the analyzed thyroid nodules. Our study identified new thyroid transcripts with altered levels in PTC using the NanoString approach. A predictive score correlation coefficient might contribute to improve the preoperative diagnosis of thyroid nodules.

## INTRODUCTION

Thyroid cancer is the most common endocrine malignancy with increasing incidence over the last decade [1]. Well-differentiated thyroid carcinomas, comprising papillary thyroid carcinoma (PTC, 85%) and follicular thyroid carcinoma (FTC, 15%), represent the vast majority of thyroid malignancies. Poorly differentiated and undifferentiated (anaplastic) thyroid carcinomas (PDTC and ATC) are less common but more aggressive [2]. Fine-needle aspiration (FNA) biopsy represents the gold standard for the preoperative diagnosis of PTC with an accuracy of 90% and is recommended for the clinical evaluation of all suspicious non-secreting thyroid nodules ≥ 1cm [3, 4]. Thyroid FNA allows the accurate preoperative recognition of thyroid malignancies, except for 30% of indeterminate cases, where the distinction between benign and malignant nodules is impossible based on cytological features. At present, genetic tests including the combination of *BRAF*^ V600E^ and *RAS* point mutations and *RET-PTC* and *PAX8-PPARγ* rearrangements are considered to provide high specificity and positive predictive value for PTC and FTC diagnosis (“rule in” tests). However, the sensitivity of these mutation analyses is low. Moreover, *RAS* or *PAX8/PPARγ* mutations are also found in a subset of follicular adenomas, therefore limiting their predictive value. In contrast, gene expression classifier tests such as Afirma have an excellent negative predictive value, with a limited specificity (“rule out” tests). The presence of the *BRAF*^V600E^ mutation in PTC is one of the best-defined prognostic markers today, demonstrating also a strong association with disease aggressiveness ([5] and references therein). Moreover, molecular biomarkers with strongly altered expression levels in PTC, including *TIMP1* [6], *c-KIT* [7], *c-MET* [8], or *TPO* (thyroperoxidase) [9] have been proposed to be predictive of PTC. Of note, the *BRAF^V600E^* mutation exhibited correlation with changes in *TIMP1* and *TPO* expression levels in PTC [6].

Our recent study has revealed that circadian clocks are operative in human thyroid tissue with altered properties in PTC and PDTC nodules. Moreover, the expression levels of core clock genes *BMAL1* and *CRY2* were altered in PTC tissue samples, as compared to normal thyroid and benign nodules [3]. Interestingly, alterations in *BMAL1* levels strongly correlated with those of *TIMP1* in the same PTC nodules. This study further underscores a tight connection between circadian clock alterations and cell malignant transformation in general, and revealed a possible role of the circadian clock, or of core clock components, in human thyroid malignancies. Moreover, it suggested an opening for employing circadian core clock components as potential diagnosis markers for thyroid malignancies [10]. In line with our findings, a recent report suggested that levels of the nuclear receptors *REV-ERBα* and *RORα* are significantly altered in PTC and that these alterations correlate with the presence of the *BRAF^V600E^* mutation [11].

In spite of substantial efforts [12–15], at present there are still no reliable preoperative tests/diagnosis markers available for patients with indeterminate thyroid FNA cytology. Therefore, the search for reliable preoperative markers of malignancy for thyroid nodules with indeterminate cytology stays of utmost clinical importance [16]. In an attempt to further characterize the involvement of the core clock machinery in thyroid malignancies and to search for additional reliable markers for PTC diagnosis, we performed comparative large-scale transcript expression analysis by probe-based NanoString, evaluated as one of the most accurate approaches for gene expression assessment [17]. Obtaining a large number of fresh-frozen postoperative biopsy samples represents a significant challenge for large-scale transcript analysis. We therefore explored an alternative approach by employing archival formalin-fixed paraffin-embedded (FFPE) samples, which are more readily available. Although formalin fixation affects nucleic acid quality, it has been recently suggested that NanoString nCounter technology allows for reliable gene expression measurements, using RNA extracted from oral squamous cell carcinoma and malignant breast tissue FFPE samples [18, 19]. We thus aimed at validating NanoString as a method of choice for FFPE thyroid sample gene expression analysis, and to apply this approach for a rationally designed search for potential biomarker candidates by comparison of transcript expression in PTC versus benign thyroid nodules.

## RESULTS

### FFPE thyroid nodule samples allow for a reliable estimation of transcript expression levels in comparison to fresh-frozen samples assessed by NanoString nCounter™ analysis

To validate the application of the NanoString nCounter™ approach for thyroid nodule sample analysis, FFPE and fresh-frozen tissue samples were obtained from 8 donors (4 benign nodules and 4 PTC in each group of samples; see Table 1 for donor characteristics). The expression levels of 34 transcripts (See Methods and Supplemental Table 1, code-set 1) were assessed in RNA samples extracted from FFPE and fresh-frozen tissues by NanoString nCounter™ technology and compared between frozen and FFPE sample groups (Supplemental Table 2). The statistical analysis revealed significant differences in 2 out of 34 transcripts in the benign and PTC groups, respectively, with the fold difference fixed at ≥ 2 and a raw *P*-value < 0.05. No transcripts showed significant difference in their false discovery rate (FDR) 5% (see Supplemental Table 2 for details). Moreover, high Pearson correlation coefficients were observed for each pair of FFPE and frozen sample (average of r^2^ = 0.84; 0.63 < r^2^ < 0.99). The results of these proof-of-principle experimental series were in good agreement with previous reports [18, 19] and strongly suggested that RNA extracted from FFPE samples represents a reliable material for NanoString analysis despite its high level of degradation.

**Table 1:** Donor characteristics and diagnosis

Code set	Case	Sex	Age, years	Operation time	Size (cm), malignant nodule	Histologic diagnosis
***PTC***						
1	1^*^	M	56	13h 40min	1.8	^I^Classical PTC, pT1b
	2	F	54	12h 50min	3.0	^II^Classical PTC, pT3pN0
	3	F	72	8h 30min	5.0	^II^Classical PTC, pT3pN1a
	4	F	63	8h 45min	4.1	^II^Classical PTC, pT3 (m)
	5^*^	M	25	8h 30min	3.2	^II^Classical PTC, pT2pN1a
	6^*^	F	51	12h 50min	2.5	^II^Classical PTC, pT3 (m) pN1a
	7^*^	F	30	8h 35min	1.5	^II^Classical PTC, pT1bpN0
2	8	M	39	13h 15min	0.9	^I^Follicular variant PTC, pT1a pN1b
	9	F	31	13h 30min	3.0	^II^Classical PTC, pT2
	10	M	26	14h 00min	3.2	^II^Mixed PTC, pT2
	11	F	64	13h 40min	2.0	^II^Tall cell PTC, pT3 pN1a
	12	F	42	10h 30min	5.0	^II^Classical PTC, pT3pN1b
	13	F	21	12h 45min	2.5	^II^Classical PTC, pT2
	14	M	79	9h 45min	2.1	^II^Classical PTC, pT3 (m)
	15	F	41	10h 55min	2.3	^II^Classical PTC, pT3 (m) pN1b
	16	F	48	9h 20min	2.0	^II^Classical PTC, pT3 pN1b
	17	F	71	9h 20min	4.0	^II^Classical PTC, pT3 pN0
***Benign***						
1	18	F	46	8h 25min		
	19	F	40	8h 40min		
	20	F	57	10h 40min		
	21^*^	F	68	9h 30min		
	22^*^	M	49	10h 30min		
	23^*^	F	47	10h 30min		
	24^*^	F	52	8h 20min		
2	25	F	50	11h 10min		
	26	F	41	9h 00min		
	27	F	45	9h 30min		
	28	F	69	9h 50min		
	29	F	62	10h 00min		
	30	F	61	12h 00min		
	31	F	66	11h 45min		
	32	F	46	10h 05min		
	33	F	45	8h 20min		
	34	F	50	11h 35min		

According to the histologic type and invasiveness, PTC samples were divided into two groups corresponding to the clinical aggressiveness:

I less aggressive PTC

II more aggressive PTC

* Cases with fresh-frozen and FFPE samples analyzed for methodology validation (see Suppl. Table 2).

### NanoString analysis of transcript expression patterns in benign and PTC thyroid nodules obtained from distinct donors

Our recent study had revealed alterations in the expression levels of the core clock transcripts *BMAL1* and *CRY2* in PTC in comparison to benign nodules and healthy tissue, as measured by qRT-PCR [3]. In order to further explore the connection between core clock gene expression levels and PTC progression and to search for additional reliable PTC preoperative diagnostics markers, we conducted the NanoString analysis of 17 PTC and 17 benign FFPE samples, obtained during thyroid surgery from distinct donors (see Table 1 for donor characteristics and postoperative diagnosis). 51 genes, comprising those related to thyroid function, core clock, cell cycle and apoptosis, were selected for analysis in benign and PTC FFPE samples (Supplemental Table 1). Several transcripts, previously demonstrated to exhibit strong expression level changes in PTC, such as *TIMP1*, *c-MET* and *c-KIT* [6, 7, 20, 21], were included for additional validation of the methodology and for the correlation analysis. Our study revealed that the levels of *TIMP1* and *c-MET* were 6-fold upregulated, while those of *c-KIT* were 11-fold downregulated in PTC (Table 3, upper part), which is in good agreement with the works of others [6, 7, 20, 21], and our own previous study [3]. Furthermore, the levels of *VEGFR1*, *PPARγ*, *TG* (thyroglobulin), *DIO2*, and *ALDH1* transcripts were moderately (2 - 5-fold) downregulated. The levels of *SLC26A4* (pendrin) and *TPO* were strongly downregulated on average, however the expression levels of both transcripts varied vastly among the samples, and therefore the changes were not statistically significant (*P*-value > 0.05). Our results are in good agreement with previous studies that have assessed these genes individually in PTC samples using immunohistochemistry and quantitative RT-PCR [22–26]. In addition to confirming previous findings, a large-scale screening by NanoString analysis allows for the combined high-precision evaluation of changes in the expression levels of a large number of transcripts in the same PTC sample.

**Table 2:** Donor characteristics and diagnosis: multinodular goiter cases

Code set	Case	Sex	Age, years	Operation time	Size (cm), malignant tumor	Histologic diagnosis
***PTC/Benign***						
1	35/36	F	38	11h 35min	3.8	^I^Follicular variant PTC, pT2 (m)
	37/38	F	79	9h 35 min	3.5	^I^Follicular variant PTC, pT2 (m)
	39/40	F	42	9h 45min	2.5	^I^Follicular variant PTC, pT2
	41/42	F	24	10h 40min	3.9	^I^Follicular variant PTC, pT2
	43/44	F	59	12h 55min	1.1	^I^Classical PTC, pT1b (m)
	45/46	M	29	8h 00min	5.5	^II^Classical PTC, pT3 pN0
	47/48	M	65	9h 05min	5.0	^II^Classical PTC, pT3
	49/50	F	31	12h 40min	1.7	^II^Classical PTC, pT3
	51/52	F	51	12h 50min	2.5	^II^Classical PTC, pT3 (m) pN1a
	53/54	F	74	10h 10min	0.9	^II^Classical PTC, pT3
	55/56	M	47	8h 00min	3.5	^II^Classical PTC, pT2 (m) pN0
	57/58	F	49	9h 30min	2.2	^II^Classical PTC, pT3 (m) pN1a
2	59/60	F	63	15h 00min	2.2	^I^Follicular variant PTC, pT2
	61/62	F	58	9h 10min	1.5	^I^Follicular variant PTC, pT1b
	63/64	F	49	8h 10min	1.3	^I^Follicular variant PTC, pT1b
	65/66	F	55	14h 00min	1.1	^I^Follicular variant PTC, pT1b
	67/68	M	63	17h 00min	3.1	^I^Follicular variant PTC, pT2
	69/70	F	66	8h 20min	3.5	^I^Classical PTC, pT2
	71/72	F	69	13h 10min	2.8	^II^Oncocytic PTC, pT2
	73/74	F	36	14h 00min	1.5	^II^Classical PTC, pT1b
	75/76	F	35	8h 20min	1.5	^II^Follicular variant PTC, pT3(m) pN1a
	77/78	F	29	8h 25min	1.3	^II^Classical PTC, pT1b
	79/80	F	76	12h 50min	1.2	^II^Classical PTC, pT1b
	81/82	F	41	13h 45min	1.1	^II^Solid PTC, pT1b

I less aggressive PTC

II more aggressive PTC

**Table 3:** Altered transcript expression in PTC samples as compared to benign counterparts from different donors

Gene	*P*-value (PTC vs benign)	*P*-value with FDR (PTC vs benign)	Fold change	Total number of samples	Number of samples with expression value > 50 (linear scale)
*Without consideration of clinical aggressiveness*					
*BCL2*	5.86 × 10^−11^	3.51 × 10^−10^	−3.17	34	34
*BMAL1*	5.56 × 10^−10^	2.50 × 10^−9^	4.44	34	34
*CHEK1*	8.88 × 10^−4^	1.45 × 10^−3^	2.97	34	32
*c-KIT*	4.60 × 10^−7^	1.38 × 10^−6^	–10.80	34	32
*c-MET*	5.15 × 10^−13^	9.27 × 10^−12^	5.96	34	34
*PPARγ*	6.33 × 10^−6^	1.42 × 10^−5^	−3.96	34	31
*TG*	2.71 × 10^−6^	6.96 × 10^−6^	−3.85	34	34
*TIMP1*	1.02 × 10^−11^	9.18 × 10^−11^	6.27	34	34
*VEGFR1*	2.42 × 10^−4^	4.36 × 10^−4^	–2.18	34	34
*ALDH1*	3.97 × 10^−8^	6.75 × 10^−7^	–5.69	20	20
*DIO2*	2.51 × 10^−3^	5.34 × 10^−3^	–3.61	20	20
*With consideration of clinical aggressiveness: more aggressive PTC (group II in Table 1)*					
*AKT2*	6.75 × 10^−5^	1.35 × 10^−4^	−2.01	32	32
*BCL2*	1.28 × 10^−10^	7.70 × 10^−10^	−3.24	32	32
*BMAL1*	1.74 × 10^−9^	7.82 × 10^−9^	4.05	32	32
*CHEK1*	1.30 × 10^−3^	1.79 × 10^−3^	3.01	32	30
*CRY2*	1.40 × 10^−8^	5.02 × 10^−8^	−2.02	32	32
*c-KIT*	3.22 × 10^−7^	9.65 × 10^−7^	−12.21	32	30
*c-MET*	2.48 × 10^−12^	4.47 × 10^−11^	5.99	32	32
*PPARγ*	1.61 × 10^−6^	3.61 × 10^−6^	−4.46	32	29
*TG*	1.14 × 10^−6^	2.92 × 10^−6^	−4.23	32	32
*TIMP1*	5.38 × 10^−11^	4.85 × 10^−10^	5.91	32	32
*VEGFR1*	7.51 × 10^−5^	1.35 × 10^−4^	−2.37	32	32
*ALDH1*	1.49 × 10^−7^	2.53 × 10^−6^	−5.64	19	19
*DIO2*	3.81 × 10^−3^	8.10 × 10^−3^	−3.65	19	19

The fold changes between the transcript expression levels in PTC and benign (B) samples were calculated as follows: **Fc (fold change) PTC/B = 2^(average log(PTC)-average log(B))^** where PTC and B are normalized expression values obtained by NanoString analysis for the respective samples.

Importantly, levels of the core clock transcript *BMAL1* exhibited a 4-fold upregulation in the studied PTC samples, which is in agreement with our recent findings [3]. Moreover, our analysis revealed for the first time that the cell-cycle related transcript *CHEK1* and the apoptosis-related transcript *BCL2* were 3 fold up- and downregulated in PTC, respectively (Table 3, upper part).

To address possible correlations between disease progression and gene expression changes, we next analyzed transcript changes solely in samples with a more aggressive form of the disease according to histologic type and invasiveness, which represented 88.2% (15 out of 17) of PTC cases in this group (see Table 3, lower part). In these PTC samples, *CRY2* and *AKT2* transcripts exhibited a 2-fold downregulation, in addition to the transcripts altered for the entire group of samples (compare upper and lower parts of Table 3). Of note, a 2-fold downregulation of *CRY2* expression was previously observed by us employing qRT-PCR in a smaller subset of samples [3]. Changes for *AKT2* in PTC samples have not been previously reported.

### NanoString analysis of transcript expression pattern in benign thyroid nodules and PTC pairs obtained from the same donors (multinodular goiter surgeries)

We next analyzed a second group of samples comprising 24 pairs of benign and PTC samples obtained from multinodular goiter surgeries. As for the first group of patients, surgeries were performed during the same time window (see Table 2 for patient characteristics and diagnosis). In addition to increasing the number of donors, comparing PTC and benign nodule samples, derived from the same donors, aimed to ensure that the observed differences in transcript expression are resulting from tumor progression solely and not from genetic background differences among the subjects. NanoString analysis of 51 transcript levels was performed in these 48 FFPE samples (24 pairs; Table 2). As demonstrated in Table 4, upper part, only *CHEK1*, *c-KIT*, *c-MET* and *TIMP1* exhibited 2 - 3-fold differences in PTC samples compared to their benign counterparts. These differences are clearly less pronounced if compared to the respective differences that we obtained in the first part of the study (compare fold changes in the upper parts of Tables 3 and 4). Of note, in contrast to the first group of analyzed PTC samples, with the majority being in the category of clinically more aggressive subtypes (88.2%), only about half (54.2 %) of the multinodular goiter PTCs were diagnosed postoperatively as aggressive (compare Table 2 to Table 1). Separate analysis of a subset of the multinodular goiter samples with more aggressive PTC subtypes revealed 2 - 4-fold upregulation in the levels of *CHEK1*, *c-MET* and *TIMP1*, and a 2-4-fold downregulation of *BCL2*, *c-KIT*, *TG* and *PPARγ* (Table 4, lower part). *BMAL1* was 1.9-fold upregulated in this subgroup of PTC samples (Table 4, lower part). These results are generally in agreement with those obtained in the first part of the study, although observed fold changes are less pronounced. This difference might be attributed to the more homogeneous genetic background between benign and PTC nodules in this group, ensuring that the differences are solely associated with PTC malignancy progression and also to the overall less aggressive form of PTC in this group.

**Table 4:** Altered transcript expression in PTC samples as compared to benign counterparts from the same donors (multinodular goiter cases)

Gene	*P*-value (PTC vs benign)	*P*-value with FDR (PTC vs benign)	Fold change	Total number of samples	Number of samples with expression value > 50 (linear scale)
*Without consideration of clinical aggressiveness*					
*CHEK1*	2.63 × 10^−4^	1.15 × 10^−3^	2.05	48	47
*c-KIT*	2.38 × 10^−6^	2.39 × 10^−5^	−3.23	48	48
*c-MET*	2.66 × 10^−6^	2.39 × 10^−5^	2.73	48	48
*TIMP1*	2.00 × 10^−3^	6.00 × 10^−3^	2.01	48	48
*With consideration of clinical aggressiveness: more aggressive PTC (group II in* Table 2)					
*BCL2*	3.08 × 10^−6^	1.38 × 10^−5^	−2.11	26	26
*BMAL1*	7.30 × 10^−4^	1.64 × 10^−3^	1.93	26	26
*CHEK1*	1.32 × 10^−3^	2.08 × 10^−3^	2.16	26	26
*c-KIT*	1.59 × 10^−6^	9.63 × 10^−6^	−4.25	26	26
*c-MET*	5.64 × 10^−11^	1.02 × 10^−9^	4.55	26	26
*PPARγ*	1.59 × 10^−3^	2.21 × 10^−3^	−2.81	26	25
*TG*	1.60 × 10^−6^	9.63 × 10^−6^	−3.03	26	26
*TIMP1*	1.22 × 10^−5^	4.38 × 10^−5^	3.38	26	26

Lastly, we performed a combined analysis of all 41 PTC samples enrolled in this study (17 in the first part and 24 in the second part), and compared those to the 17 benign samples from the first part of the study (Table 5), resulting in a total of 58 samples. The 24 benign nodules from the second group of samples were not included in this analysis, to satisfy independent samples criteria for statistical analysis and to ensure homogenous comparison among the 58 samples derived from different donors. Most of the transcript changes, identified in the first part of the study, were also confirmed by this combined analysis of 41 PTC samples (compare Table 3 and 5). Interestingly, *VDR* appeared 3-fold upregulated in the combined PTC group, in good agreement with recent work [27]. Of note, even though the fold-change of the core clock transcripts *CRY2, PER2* and *PER3* was below the cut-off value (2-fold change) in this group of PTC samples, a tendency towards a statistically significant 1.5 – 2-fold downregulation was observed, consistent with the qRT-PCR results obtained by us previously [3]. Taking into account the clinical characteristics of the PTC cases, an analysis performed on the more aggressive cases only (Table 5, lower part) confirmed significant alterations in the levels of all transcripts appearing in Table 3, except for *AKT2* and *CRY2*, which were now downregulated slightly below 2-fold and thus qualified as non-significant. Importantly, the 3-fold upregulation of *BMAL1* and *CHEK1*, and the 3-fold downregulation of *BCL2* in the overall PTC group provided further validation of these genes as potential new biomarkers for PTC.

**Table 5:** Altered transcript expression in PTC versus benign samples (combined PTC samples)

Gene	*P*-value (PTC vs benign)	*P*-value with FDR (PTC vs benign)	Fold change	Total number of samples	Number of samples with expression value > 50
Without consideration of clinical aggressiveness					
*BCL2*	1.86 × 10^−6^	1.42 × 10^−5^	−2.21	58	58
*BMAL1*	3.15 × 10^−6^	1.42 × 10^−5^	2.87	58	58
*CHEK1*	5.48 × 10^−6^	1.97 × 10^−5^	2.97	58	56
*c-KIT*	2.43 × 10^−6^	1.42 × 10^−5^	−5.64	58	56
*c-MET*	5.41 × 10^−8^	9.74 × 10^−7^	3.48	58	58
*PPARγ*	1.64 × 10^−3^	2.95 × 10^−3^	−2.55	58	54
*TG*	6.38 × 10^−4^	1.28 × 10^−3^	−2.39	58	58
*TIMP1*	1.34 × 10^−5^	3.45 × 10^−5^	3.39	58	58
*ALDH1*	2.66 × 10^−3^	1.97 × 10^−2^	−2.55	32	32
*VDR*	2.38 × 10^−3^	2.11 × 10^−2^	3.15	26	25
*With consideration of clinical aggressiveness: more aggressive PTC (group II in Tables 1,2)*					
*BCL2*	2.33 × 10^−9^	2.10 × 10^−8^	−2.70	45	45
*BMAL1*	1.15 × 10^−7^	3.46 × 10^−7^	3.49	45	45
*CHEK1*	9.81 × 10^−6^	2.21 × 10^−5^	3.10	45	43
*c-KIT*	7.07 × 10^−8^	2.55 × 10^−7^	−7.82	45	43
*c-MET*	1.30 × 10^−12^	2.35 × 10^−11^	4.81	45	45
*PPARγ*	2.30 × 10^−5^	4.60 × 10^−5^	−3.56	45	41
*TG*	2.03 × 10^−7^	5.22 × 10^−7^	−3.52	45	45
*TIMP1*	2.05 × 10^−8^	9.24 × 10^−8^	4.79	45	45
*ALDH1*	2.63 × 10^−4^	2.30 × 10^−3^	−3.26	25	25
*DIO2*	2.24 × 10^−2^	4.23 × 10^−2^	−2.41	25	25
*VDR*	2.85 × 10^−3^	2.28 × 10^−2^	3.36	20	19

41 PTC samples enrolled in the study (17 PTC samples from the first cohort (Table 1), and 24 PTC from the second cohort (Table 2)) were compared to 17 benign nodules from the first cohort (Table 1).

### Alterations of *BMAL1, CHEK1, c-MET, c-KIT* and *TIMP1* expression levels in PTC exhibit pair-wise correlations

**Figure 1:**
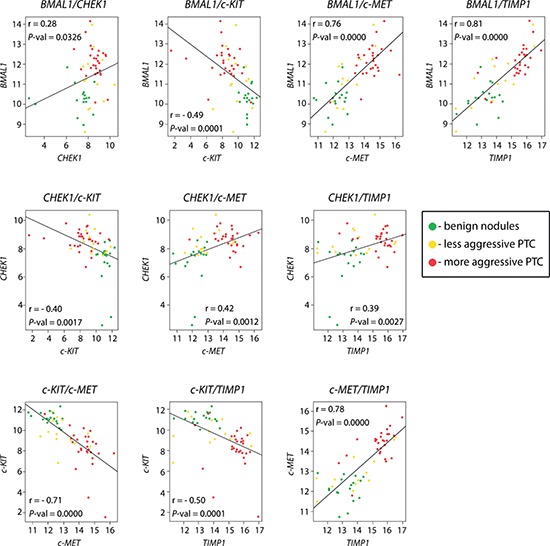
Pair-wise correlations between BMAL1, CHEK1, TIMP1, c-KIT and c-MET transcript changes in PTC Pearson correlation analysis revealed pair-wise correlations of gene expression in PTC (combined samples from 1^st^ and 2^nd^ cohorts, Tables 1 and 2) and benign (1^st^ cohort, Table 1) samples. The correlation strength was based on Evans classification (see Methods for details), with a coefficient r value < 0.20 reflecting very weak correlation; 0.20 – 0.39 – weak; 0.40 – 0.59 – moderate; 0.60 – 0.79 – strong; and > 0.80 - very strong. The dots at each graph are representing normalized respective gene expression values.

Given that the NanoString approach allows for the assessment of numerous transcript levels within the same samples, we next addressed possible correlations among transcript changes upon PTC. Pair-wise correlation analysis was performed between transcripts, which exhibited the most marked alterations in both studied cohorts. Analysis of the combined set of 58 PTC samples enrolled in this study (Tables 1, 2) revealed that *c-KIT* expression levels inversely correlated with all the other transcript changes, exhibiting strong correlation with *c-MET* and moderate correlation with *BMAL1, CHEK1* and *TIMP1* (Figure 1). *BMAL1* level changes were strongly positively correlated with those of *c-MET* and *TIMP1* and weakly with *CHEK1* (Figure 1). In line with these findings, our recent study suggested a positive correlation between *BMAL1* and *TIMP1* transcript levels, assessed by qRT-PCR in the same PTC samples [3]. *CHEK1* exhibited a weak to moderate positive correlation with *c-MET* and *TIMP1*, while *c-MET* and *TIMP1* exhibited a strong positive correlation among each other in the same PTC samples (Figure 1). Our joint NanoString analysis of *BMAL1, CHEK1, c-MET, c-KIT* and *TIMP1* levels in the same PTC samples suggest that expression level changes in these transcripts might be correlated. Therefore, this group of transcripts represents a plausible cluster of markers whose collective changes are associated with PTC development and which might be potentially predictive for PTC diagnosis.

### *BRAF^V600E^* mutation analysis in PTC samples

The *BRAF*^V600E^ mutation is associated with PTC in 40–70% of cases [9]. Although the benefit of assessing the *BRAF*^V600E^ mutational status preoperatively, as a method to determine the extent of thyroidectomy and lymph node dissection, remains uncertain, it might be considered useful in order to support the preoperative diagnosis, especially in cases that are potentially malignant (Bethesda category V). In an attempt to acquire additional valuable characteristics of the PTC nodules, analyzed in this study by NanoString, we conducted a *BRAF^V600E^* mutation analysis in the same samples. As listed in Table 6, 81% (13/16) of PTC samples from the first group, diagnosed as classical PTC, exhibited the *BRAF^V600E^* mutation, which is in agreement with a previous report [6]. In the multinodular goiter PTC cases only 12 PTC samples were of the classical type, with 67% (8/12) exhibiting the *BRAF^V600E^* mutation (Table 6, lower part).

**Table 6:** *BRAF* exon 15 mutation analysis

Group	Case	BRAF exon 15		Histologic diagnosis
		PROT	DNA	
**PTC samples (Table 1)**	1	p.V600E	c.1799T>A	^I^Classical PTC, pT1b
	2	WT	WT	^II^Classical PTC, pT3pN0
	3	p.V600E	c.1799T>A	^II^Classical PTC, pT3pN1a
	4	p.V600E	c.1799T>A	^II^Classical PTC, pT3 (m)
	5	p.V600E	c.1799T>A	^II^Classical PTC, pT2pN1a
	6	p.V600E	c.1799T>A	^II^Classical PTC, pT3 (m) pN1
	7	p.V600E	c.1799T>A	^II^Classical PTC, pT1bpN0
	8	p.V600E	c.1799T>A	^I^Follicular variant PTC, pT1a pN1b
	9	p.V600E	c.1799T>A	^II^Classical PTC, pT2
	10	p.V600E	c.1799T>A	^II^Mixed PTC, pT2
	11	p.V600E	c.1799T>A	^II^Tall cell PTC, pT3 pN1a
	12	WT	WT	^II^Tall cell PTC, pT3 pN1a
	13	WT	WT	^II^Classical PTC, pT2
	14	not accessible		^II^Classical PTC, pT3 (m)
	15	p.V600E	c.1799T>A	^II^Classical PTC, pT3 (m) pN1b
	16	p.V600E	c.1799T>A	^II^Classical PTC, pT3 pN1b
	17	p.V600E	c.1799T>A	^II^Classical PTC, pT3 pN0
**Multinodular goiter PTC samples** (Table 2)	35	WT	WT	^I^Follicular variant PTC, pT2 (m)
	37	WT	WT	^I^Follicular variant PTC, pT2 (m)
	39	WT	WT	^I^Follicular variant PTC, pT2
	41	WT	WT	^I^Follicular variant PTC, pT2
	43	WT	WT	^I^Classical PTC, pT1b (m)
	45	p.V600E	c.1799T>A	^II^Classical PTC, pT3 pN0
	47	WT	WT	^II^Classical PTC, pT3
	49	p.V600E	c.1799T>A	^II^Classical PTC, pT3
	51	p.V600E	c.1799T>A	^II^Classical PTC, pT3 (m) pN1a
	53	p.V600E	c.1799T>A	^II^Classical PTC, pT3
	55	p.V600_K601>Ep.R603Q	c.1799_1801delTGAWT	^II^Classical PTC, pT2 (m) pN0
	57	WT	WT	^II^Classical PTC, pT3 (m) pN1a
	59	WT	WT	^I^Follicular variant PTC, pT2
	61	WT	WT	^I^Follicular variant PTC, pT1b
	63	WT	WT	^I^Follicular variant PTC, pT1b
	65	WT	WT	^I^Follicular variant PTC, pT1b
	67	WT	WT	^I^Follicular variant PTC, pT2
	69	WT	WT	^I^Classical PTC, pT2
	71	WT	WT	^II^Oncocytic PTC, pT2
	73	p.V600E	c.1799T>A	^II^Classical PTC, pT1b
	75	p.V600E	c.1799T>A	^II^Follicular variant PTC, pT3(m) pN1a
	77	WT	WT	^II^Classical PTC, pT1b
	79	p.V600E	c.1799T>A	^II^Classical PTC, pT1b
	81	WT	WT	^II^Solid PTC, pT1b

I less aggressive PTC

II more aggressive PTC

Mutation nomenclature is based on the COSMIC data base (Catalogue of Somatic Mutations in Cancer) Sanger Institute: WT– BRAF wild type; pV600E, pV600_K601>E, p. R603Q – mutation at BRAF protein level; c.1799T>A, c.1799_1801delTGA, c1808G>A – mutation at BRAF nucleotide level.

### Predictive score for PTC diagnosis based on *BMAL1, CHEK1, TIMP1, c-MET*, and *c-KIT* combined expression level changes and on *BRAF^V600E^* mutation analysis

In an attempt to correlate the degree of expression level changes of *BMAL1*, *CHEK1*, *TIMP1, c-MET* and *c-KIT* with the histological diagnosis, we aimed at establishing a gene expression-based predictive score, taking into account collective biomarker changes [28, 29]. Along with such a gene expression-based score, the *BRAF^V600E^* mutation status was employed as an additional parameter for establishing a correlation with the postoperative clinical evaluation.

A final predictive score was established for each biological sample, based on the expression levels of five distinctive genes *BMAL1, CHEK1, c-KIT, c-MET* and *TIMP1,* which exhibited stable changes in PTC and correlated among each other using the Linear Prediction Score (LPS; for details see Supplemental Methods; [29]). To test the performance of the score, a receiver operating characteristic (ROC) analysis was performed, and ROC curves were established (see Supplemental Figure 1 and Supplemental Methods).

**Figure 2:**
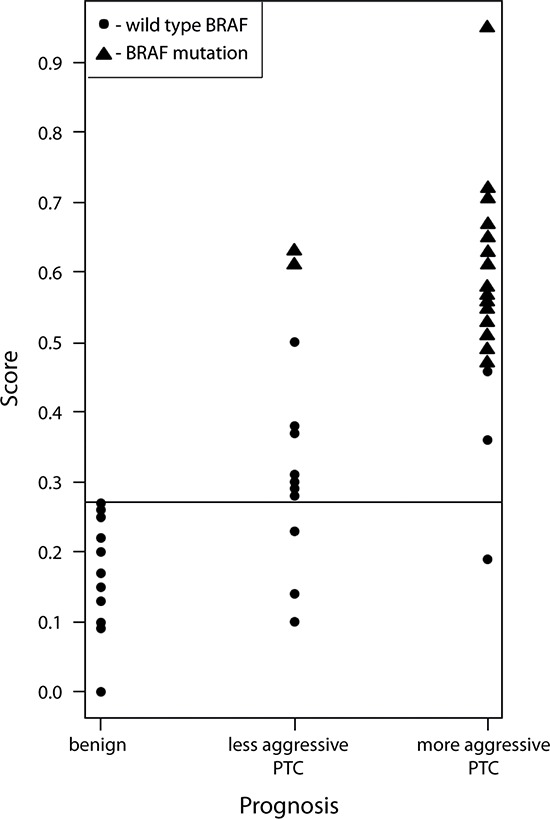
Scatter plot of gene expression-based predictive scores correlated to PTC biological aggressiveness and BRAF mutation status The gene expression-based score for benign and PTC samples was calculated based on joint expression levels of *BMAL1, CHEK1*, *c-KIT, c-MET* and *TIMP1* transcripts. Thus obtained score values were plotted for three clusters: benign, less aggressive PTC and more aggressive PTC, and allowed a clear distinction between these groups. *BRAF^V600E^* mutation was strongly associated with samples characterized by the highest predictive score values and with more aggressive PTC diagnosis.

Our results indicate that at the threshold = 0.27, based on empirical curve analysis (Supplemental Figure 1), our predictive score discriminates PTC from benign cases with 90% sensitivity and 100% specificity in the validation set. The thus obtained scores were plotted for each sample of the three sample groups and divided according to their clinical characteristics into: benign, less aggressive PTC and more aggressive PTC (Figure 2). Among the total of 58 samples, 4 samples only (7%) were misclassified according to the expression-based score analysis. In addition, striking correlations were observed between higher scores and PTC aggressiveness (Figure 2). Turning to *BRAF^V600E^* mutation mapping in the same samples, 19 out of 27 PTC samples with more aggressive histological type bore the *BRAF^V600E^* mutation, while in the less aggressive histological group only 2 out of 13 samples had the mutation (samples marked as triangles at Figure 2; Supplemental Table 3). Thus established gene expression-based score, combined with *BRAF^V600E^* mutation analysis, might help to distinguish between benign and PTC samples, especially for more aggressive (classical PTC) cases. Although these results strongly suggest a predictive value for PTC diagnosis, follow-up studies with a higher number of samples will be required to estimate the here proposed coefficient validity.

## DISCUSSION

### RNA quantification in archival FFPE human thyroid nodule samples using NanoString analysis

NanoString nCounter^TM^, a color-coded probe-based method, has been reported to attain superior gene expression quantification results in comparison to qRT-PCR [19, 30]. A major limitation of RNA quantification studies performed in fresh post-operative thyroid nodules, including our own recent study [3], is the low number of available samples, in particular for the malignant nodules. To overcome this, we turned to much more readily available archival FFPE material. Our analysis suggests that FFPE samples provide a reliable alternative for NanoString analysis as compared to fresh-frozen tissue biopsy samples (Supplemental Table 2). In summary, our study validates the NanoString method for assessing transcript expression in FFPE human thyroid nodule samples and is in good agreement with recent work performed with FFPE samples from different tissues [18, 19]. Strong advantages of the NanoString approach are high precision due to direct probe hybridization and collective assessment of a large number of transcripts in the same sample. The use of FFPE samples greatly increases the available material and thus statistical relevance of the study. However, the significant costs of NanoString screening might represent a serious drawback if employed for routine diagnosis. Therefore, alternative cost-efficient methods for the screening of a large number of samples, for example by multiplex qPCR analysis of the newly identified subset of biomarkers, will be more appropriate for clinical applications.

### Altered transcript expression in human PTC: identifying new and confirming previously reported potential biomarkers by NanoString analysis

The essential cell cycle component *CHEK1* and the apoptosis related gene *BCL2,* previously reported to be associated with a number of malignancies [31–34], were identified for the first time by our screening to exhibit significant changes in their expression levels in PTC (Tables 3–5). *CHEK1* was significantly upregulated in all PTC samples, however it did not exhibit a clear correlation with PTC aggressiveness and displayed only a weak to moderate correlation with other analyzed genes (Tables 3–5, Figure 1). Providing further insights into the causality and molecular mechanisms of these alterations in cell cycle, apoptosis, as well as core clock key components with thyroid malignancy development might be of great interest (see below).

In good agreement with a number of previous studies [6–8], NanoString analysis of two groups of samples (total number of 41 benign and 41 PTC samples) revealed that *TIMP1, c-KIT* and *c-MET* exhibited highly significant alterations in PTC with pronounced fold changes and low variability among the samples (Tables 3–5). The role of these transcripts in oncogenic transformation in general and their association with human PTC has been well established [35–37]. Of note, we observed pair-wise correlations between these genes, namely *TIMP1* and *c-MET* exhibited strong positive correlation, while *c-KIT* was strongly and moderately inversely correlated with *c-MET* and *TIMP1*, respectively (Figure 1). Consistently with previous studies, these transcript level changes correlated with the presence of the *BRAF^V600E^* mutation (Figure 2; [6]).

We consider the multinodular goiter samples the most reliable ones for studying gene expression changes upon thyroid malignancies, as differences between benign and PTC nodules in this group are attributed only to tumor progression. Of note, the newly identified PTC-associated markers *CHEK1* and *BCL2* were significantly altered in a subgroup with more severe histological prognosis (Table 4, lower part). *C-KIT, c-MET* and *TIMP1* exhibited the strongest alterations in these samples. Since *PPARγ* and *TG* exhibited at least a 3-fold downregulation and little variability in multinodular goiter PTC samples, compared to their benign counterparts from the same subjects (Table 4), these genes might represent additional valuable candidates for the here presented combined predictive score. *TPO*, previously proposed to be predictive of PTC and associated with the *BRAF^V600E^* mutation status [9], indeed showed a strong downregulation in the first group of samples albeit its high variability. Strikingly, its mRNA levels did not change significantly in the multinodular goiter sample group. The same was true for *SLC26A4,* making the predictive value of these two genes for PTC rather problematic.

### Correlation between circadian clock alterations and human PTC development

Our study has validated the significant upregulation of the core clock gene *BMAL1* in PTC samples, previously shown by us in a smaller subset of samples as strongly upregulated in PTC and FTC [3]. Based on the comparison of 41 pairs of PTC and benign nodules in this study, *BMAL1* expression reliably increased about 3-fold (1.93 – 4.4-fold in different groups of samples; Tables 3–5). The fold increase of the *BMAL1* transcript was lower than previously observed, which might be due to the less aggressive character and the higher number of PTC cases enrolled in this study. However, *BMAL1* upregulation was observed in all of the analyzed samples, with low variability among the samples (*P*-value < 0.05 for all the groups; Tables 3–5), making this alteration highly significant. Additionally, the core clock component *REV-ERBα* exhibited a 2 - 3-fold upregulation in PTC in both cohorts of analyzed samples; however, the variability was extremely high for this transcript (*P*-value > 0.05) and therefore it was not considered significant. This result is highly consistent with our previous analysis [3], where *REV-ERBα* exhibited a tendency of being 2-fold upregulated, which was defined as non-significant due to high variability among the samples. Of note, *REV-ERBα* levels were recently reported to be significantly upregulated in PTC [11]. This discrepancy regarding *REV-ERBα* between our results and the work by others might be attributed to the differences in PTC sampling and/or data analysis. Finally, we observed a non-significant but stable tendency in PTC for the downregulation of *CRY2* (average of 1.9-fold; P-value < 0.05; Table 3), *PER2* and *PER3* (1.4-fold downregulation; *P*-value < 0.05), again being consistent with our own recent observations [3]. By contrast, *DBP, RORα, CRY1, CLOCK* and *PER1* transcripts, assessed in the same samples, did not exhibit any alterations in PTC (not shown). In summary, by NanoString analysis of 41 pairs of benign and PTC samples we further validated our recent findings on expression level changes of certain core clock components in human PTC, including the significant upregulation of *BMAL1*.

Of note, *BMAL1* transcript levels showed a strong positive correlation with those of *TIMP1* and *c-MET*, and were moderately inversely correlated with *c-KIT* within the same PTC samples (Figure 1; [3]). These correlations further underscore a potential link between *BMAL1* expression and possibly function, as well as thyroid tumor progression, and strongly argue that the observed correlations might have functional significance. Of note, *CHEK1,* identified for the first time by this study as significantly upregulated in PTC, has been previously found to be regulated by *BMAL1* [38]. We are therefore not only suggesting *CHEK1* as a potential new biomarker for PTC but are also further confirming the connection between the circadian clock, cell cycle regulation, and malignant transformation.

There is accumulating evidence on the important role of core clock components for cell cycle progression and timing of cell division [39]. The circadian oscillator gates cytokinesis to defined time windows in *in vitro* cultured fibroblasts and regulates key components of the cell cycle, such as *WEE1*, *CCNB1* and *CDC2* in mouse liver cells *in vivo* (reviewed in [10]). Of note, *PER2* has been suggested to play a key role as tumor suppressor by regulating DNA damage response pathways [40]. This finding is however in controversy with more recent study outcomes, demonstrating that deficiency in either *Per1* or *Per2* genes does not make mice more tumor-prone [52], suggesting that the role of the PER proteins as bona fide tumor suppressors needs to be reevaluated. *BMAL1* has been recently demonstrated to play an essential role in ROS homeostasis, oxidative stress response [41, 42], cell cycle control (reviewed in [43]), senescence [44] and mTOR signaling [45]. All these pathways are disrupted in various malignancies and are implicated in cancer development; the observed changes in *BMAL1* expression in thyroid tumors may result in disturbances in these pathways. *BMAL1* knockout mice exhibited reduced life span and premature aging [41]. Taking together, these findings and data presented here support the important role of *BMAL1* in the development of thyroid malignancies. At present, we cannot discriminate whether these changes in core clock components are important for the process of malignant transformation, or rather represent an adaptation to the transformation itself. It will be of interest to test this hypothesis by manipulating the expression levels of *BMAL1* in human primary thyrocytes, derived from benign or malignant nodules, with subsequent analysis of their oncogenic properties. Exploring the potential role of circadian clock components in the malignant transformation of human thyroid nodules represents an important step forward in our understanding of the molecular link between clock function, thyroid tissue physiology and pathophysiology of malignant nodules. Moreover, providing new insights into the role of the circadian clock in human thyroid malignancies might have important clinical perspectives, as discussed below.

### Towards a reliable correlation coefficient for PTC diagnosis: combined gene expression-based score and *BRAF* mutation analysis

Importantly, we established in this study a correlation coefficient between changes in the collective expression levels of BMAL1, CHEK1, TIMP1, c-KIT and c-MET, and the *BRAF^V600E^* mutation status and combined this with clinical evaluation obtained after surgery. Such generated predictive score exhibited 90% sensitivity and 100% specificity (Supplemental Figure 1; Figure 2; Supplemental Table 3). A previous study [29], which used a similar approach for establishing a linear predictive score, obtained 88% specificity based on 27 gene expression levels in a cohort of 109 subjects for the diagnosis of diffuse large B-cell lymphoma. The specificity value obtained in this study is well comparable to the here established score characteristics. Even though our predictive score is only indicative at this point and demands rigorous confirmation in follow-up studies, it will help if confirmed to increase the reliability of preoperative PTC diagnosis.

The next important challenge will be to apply a similar screening approach for FTC nodules, representing the group with the highest number of suspicious nodules, without clear preoperative diagnosis. We recently provided evidence [3] that human thyrocyte oscillator changes, observed upon PTC, were even more remarkable in PDTC in *in vitro* cultured primary cells, suggesting that in synchronized primary thyrocytes clock alterations might become more pronounced during malignancy progression. Thus, further analysis of core clock components in FTC and PDTC biopsy samples by NanoString analysis, as was done in this study for PTC, will shed additional light on the connection between thyroid malignancy progression and changes in core clock components. Moreover, exploring the implication of thus identified markers for PTC, and possibly FTC, for preoperative analysis using FNA material would be an additional important step forward towards improving the preoperative diagnosis of suspicious thyroid nodules. Indeed, given that the preoperative diagnosis of malignant thyroid nodules is still far from providing reliable results in numerous cases of suspicious or indeterminate nodules [46], it is of highest importance to launch a prospective study that validates the application of changes in core clock, cell cycle and thyroid function gene expression levels. In combination with the *BRAF^V600E^* mutation status and clinical examination, such a predictive score will allow for more reliable preoperative diagnosis of thyroid carcinomas.

## METHODS

### Study participants and thyroid tissue sampling

FFPE samples from benign and PTC human thyroid nodules were obtained from the archive of the Pathology Department, Geneva University Hospital. Donor characteristics are summarized in Table 1 (benign and PTC nodule samples from different donors) and Table 2 (benign and PTC nodules obtained from the same donors, multinodular goiter surgery cases). Samples from surgeries performed in the time window between 8 AM and 2 PM were chosen, with two exceptions (59/60 and 67/68, Table 2). Malignant tumors were classified by histopathological analysis according to the World Health Organization Classification of Thyroid Tumors [50] and staged according to the AJCC Cancer Staging Manual 7^th^ ed. In cases where fresh-frozen and FFPE samples were compared, one part of the obtained thyroid tissue was deep-frozen and kept for tissue transcript analysis, the other part was processed to establish FFPE samples. Fresh thyroid tissue samples (1 cm^3^) were obtained from patients undergoing thyroidectomy for thyroid cancer or a suspicious nodule. Written informed consent was obtained from each patient and the study protocol was approved by the local Ethics Committee (CER 11–014).

### RNA extraction from fresh-frozen and FFPE samples

Frozen tissue biopsies were homogenized using a Polytron homogenizer (Kinematica AG), and total RNA was extracted using the RNA spin II kit (Macherey-Nagel) according to manufacturer's instructions. For RNA extraction from FFPE samples 3 μm thick tissue sections were deparaffinized in xylol, proteins were digested overnight, and RNA was subsequently extracted using the High Pure miRNA isolation kit (Roche) according to the manufacturer's instructions.

### Gene expression quantification using multiplexed, color-coded probe pairs (NanoString nCounter^TM^)

51 candidate genes were selected for analysis, including genes involved in thyroid function, circadian clock, cell cycle, and apoptosis, based on our own previous study and on literature search. Probes were designed and synthesized by NanoString nCounter™ technologies (Supplemental Table 1). 200 – 400 ng of total RNA, extracted from fresh-frozen or FFPE samples, were hybridized with multiplexed NanoString probes, as described in [17]. Background correction was made by subtracting the mean + 2 SD from the raw counts obtained with negative controls. Values <1 were fixed to 1 to avoid negative values after log transformation. Counts for target genes were then normalized with the geometric mean of three house-keeping genes (*HPRT1*, *RLP13A* and *ACTB*) selected as the most stable ones using the geNorm algorithm [47]. Normalized data were log2 transformed for further analyses.

### DNA extraction and *BRAF* mutation PCR analysis

For analysis of the *BRAF^V600E^* mutation 3 μm thick FFPE tissue sections were deparaffinized in xylol, proteins were digested and DNA was subsequently extracted in the QIAcube using the QIAamp DNA FFPE Tissue Kit (QIAGEN). PCR was performed as previously described [48], using the following primers: forward 5′aaactcttcataatgcttgctctg3′ and reverse 5′ggccaaaaatttaatcagtgga3′PCR products were separated on an agarose gel, purified using the peqGold gel extraction kit (Peqlab) and sequenced in a capillary automatic sequencer (Applied Biosystems 96-cappilary 3730xl).

### Statistical analysis

To assess differences in gene-expression values between the different groups, ANOVA tests with contrasts (significant *P*-values < 0.05) were performed using Partek Genomics Suite (http://www.partek.com). *P*-values were corrected for multiple testing by use of the false-discovery rate (FDR) method of Benjamini and Hochberg [49]. A conservative significance threshold of 5% FDR associated with a fold change value of 2 or more was applied (see Supplementary Methods for more details on the statistical analysis). A merge of the results obtained in two independent NanoString runs was performed as described in Supplemental methods. Pair-wise Pearson correlation analysis was applied to test correlation between gene expression data obtained by NanoString. Correlation strength was interpreted as proposed by Evans [51].

## SUPPLEMENTARY METHODS


