# Supplementary material for: Identification of new biomarkers for human papillary thyroid carcinoma employing NanoString analysis
Source: Oncotarget. 2015 Mar 26;6(13):10978–93. doi: 10.18632/oncotarget.3452 (PMC4484433; doi:10.18632/oncotarget.3452)
Supplement: Supplementary file 1 [file oncotarget-06-10978-s001.pdf]

SUPPLEMENTARY METHODS

Merge of the results obtained in two independent NanoString analyses

To normalize the results obtained in two Nanostring experiments (code-sets 1 and 2), first, normalization with spiked positive controls was performed [1]. A normalization factor (NF) (Vandesompele et al., 2002) was calculated for each sample and the counts were corrected:

$$\mathbf{NF}_{\text{sample}} = \frac{\text{sum of positive controls}_{\text{sample}}}{\text{reference}}$$

$$\textbf{Corrected count}_{\text{sample/gene}} = \text{raw count}_{\text{sample/gene}} \times \text{NF}_{\text{sample}},$$

where:

reference –is the sum of positive controls of the sample (the summed value is close to the average for all samples).

Then code-set calibration was implemented using four samples (2 benign and 2 PTC samples number 42 and 46 from multinodular goiters) represented in both code-sets. For each gene in common (×21), a calibration factor (CF) was calculated:

$$\mathbf{CF}_{\text{gene}} = \text{mean of } \frac{\text{corrected count}_{\text{gene sample code-set1}}}{\text{corrected count}_{\text{gene sample code-set2}}}$$

The results from code-set 2 were adjusted to code-set 1 using CF as follows:

$$\textbf{Calibrated count}_{\text{sample code-set 2/gene}} = \text{corrected count}_{\text{sample/gene}} \times \text{CF}_{\text{gene}}$$

Gene score data analysis

Predictive score for each biological sample was based on the combined expression levels of chosen five distinction genes *BMAL1, CHEK1, c-KIT, c-MET* and *TIMP1* exhibiting stable changes in PTCs. To ensure the reproducibility of our model, we equally divided 17 benign (Table 1) and 41 PTC (Tables 1–2) samples into training and validation sets by computer-based randomization (Supplementary Table 3; [2, 3]). The training set was used to create and optimize the gene-expression score, while the validation set allowed testing the model and evaluating its significance. Using the training set and *t*-test analysis we identified the genes that were differentially expressed in the benign and PTC samples. Five genes with high fold-change expression and high significance (*P-value +5% FDR* < 0.05) were chosen for the gene-expression score calculation. The group of five distinction genes includes *BMAL1, CHEK1, c-KIT, c-MET* and *TIMP1*. To calculate PTC prediction score, first the expression levels of distinction genes were combined to calculate a Linear Predictor Score (LPS) [4] for each sample (X) in the training set:

$$\text{LPS(X)} = \Sigma_j a_j X_j$$

Where:

X_j –a gene expression value of gene j

a_j –a scaling factor, whose value depends on the degree, to which each gene discriminates in the subgroup. The scaling factors were chosen to be the t-statistics generated by a *t*-test for the difference in expression between PTC and benign subgroups.

To obtain the PTC prediction score comprised between 0 and 1, the following score transformation was performed: if LPS was less than 100, it was set to 100; if LPS was greater than 200, it was set to 200. The final PTC prediction score values were calculated as follows:

$$\text{Score (X)} = (\text{LPS(X)} - 100)/100$$

A similar procedure of PTC prediction score calculation was used for validation set. The score values for training and validation set samples are shown in Supplementary Table 3.

To test the performance of the score, a receiver operating characteristic (ROC) analysis with calculation of the ROC area under the curve (AUC) statistic was performed [2, 3, 5]. The ROC curve (Supplementary Figure 1) gives sensitivity and specificity of a certain test in function of the cut off value: the better the diagnostic test, the nearer the curve reaches to the top left corner, indicating 100% sensitivity and specificity.

SUPPLEMENTARY FIGURE AND TABLES

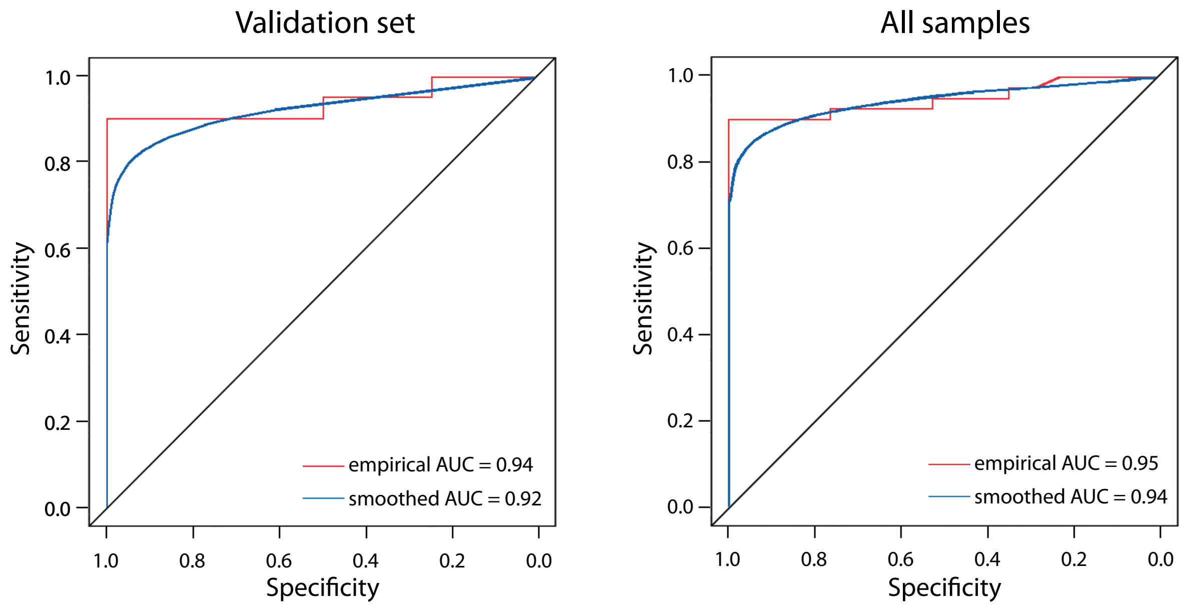

Supplementary Figure S1: ROC analysis. ROC analysis with calculation of the area under the curve (AUC) statistic was performed to test in validation set (left) and all samples (right). Binormal smoothing iteration was performed to show continuous ROC curves. At the threshold 0.27 PTC diagnostic score discriminates PTC from benign cases with 90% sensitivity and 100% specificity in the validation set (left) and all sample analysis (right).

Supplementary Table S1: NanoString probes design

Gene	Accession	Target Region	Gene	Accession	Target Region
AKT1[1]	NM_005163.2	1773–1872	*PER2*[1,2]	NM_022817.2	986–1085
AKT2[1,2]	NM_001626.2	1451–1550	*PER3*[1,2]	NM_016831.1	1076–1175
ALDH1[2]	NM_000689.4	1161–1260	*PIK3CA*[1]	NM_006218.2	2446–2545
BCL2[1,2]	NM_000657.2	6–105	*PIK3CB*[1]	NM_006219.1	2946–3045
BMAL1[1,2]	NM_001030272.1	841–940	*PLEKHA7*[1]	NM_175058.4	4137–4236
BRAF[1]	NM_004333.3	566–665	*PPARγ*[1,2]	NM_015869.3	1036–1135
CCND1[2]	NM_053056.2	691–790	*PTEN*[2]	NM_000314.3	1676–1775
CDH1[2]	NM_004360.2	536–635	*Rev-Erbα*[2]	NM_021724.3	1081–1180
CDK1[1]	NM_001786.4	179–278	*RORα*[2]	NM_134261.2	1716–1815
CDKN1A[2]	NM_000389.2	1976–2075	*SIRT1*[1]	NM_012238.4	841–940
CHEK1[1,2]	NM_001114121.1	2226–2325	*SLC5A5*[2]	NM_000453.2	3459–3558
CLOCK[1]	NM_004898.2	2351–2450	*SLC26A4*[2]	NM_000441.1	1711–1810
CRY1[1,2]	NM_004075.3	1376–1475	*SOX9*[2]	NM_000346.2	2136–2235
CRY2[1,2]	NM_001127457.1	3326–3425	*TG*[1,2]	NM_003235.4	6499–6598
CXCR4[1]	NM_003467.2	1336–1435	*TIMP1*[1,2]	NM_003254.2	330–429
DBP[2]	NM_001352.2	1051–1150	*TPO*[2]	NM_175719.3	297–396
DDIT3[1,2]	NM_004083.4	41–140	*TSHR*[1,2]	NM_001018036.2	736–835
DIO2[2]	NM_013989.3	5076–5175	*TP53*[1]	NM_000546.2	1331–1430
EGFR[1]	NM_201282.1	361–460	*UCP1*[2]	NM_021833.4	551–650
IQGAP1[1]	NM_003870.3	821–920	*VDR*[1]	NM_000376.2	4386–4485
c-KIT[1,2]	NM_000222.1	6–105	*VEGFR1*[1,2]	NM_002019.4	531–630
MCM2[2]	NM_004526.2	2946–3045	*WEE1*[2]	NM_003390.3	1226–1325
c-MET[1,2]	NM_000245.2	406–505	*House-keeping genes*		
MYC[1]	NM_002467.3	1611–1710	*ACTB*[1,2]	NM_001101.2	1011–1110
NFIL3[2]	NM_005384.2	1796–1895	*B2M*[1]	NM_004048.2	26–125
NTRK1[1]	NM_001012331.1	1366–1465	*EEF1A1*[2]	NM_001402.5	791–890
PDGFRA[1,2]	NM_006206.4	1388–1487	*GAPDH*[1]	NM_002046.3	973–1072
PDGFRB[1]	NM_002609.3	809–908	*HPRT1*[1,2]	NM_000194.1	241–340
PER1[1,2]	NM_002616.2	4366–4465	*RPL13A*[1,2]	NM_012423.2	721–820

[1]Code-set 1, [2]Code-set 2

Supplementary Table S2: Thirty four transcripts assessed by NanoString analysis exhibit comparable levels in FFPE and fresh-frozen samples obtained from the same donors

	Case[1]	Number of significantly different transcripts[2] (at 5% + FC ≥ 2)[3]	Number of significantly different transcripts (at FDR 5% + FC ≥ 2)[4]
Benign	21<br>22<br>23<br>24	2 (*PDGFRB*, *PDGFRA*)	0
PTC	1<br>5<br>6<br>7	2 (*VEGFR1*, *PPARG*)	0

[1]Donor characteristics are presented in Table 1 (subjects labeled with *);
[2]Transcript list and probes design are presented in Supplementary Table 1, code-set 1;
[3]Number of significantly different transcripts (at 5% + FC ≥ 2)" corresponds to raw-*P*-value <0.05 and fold-change in gene expression ≥ 2;
[4]Number of significant genes (at FDR 5% + FC ≥ 2) corresponds to *P*-value with False Discovery Rate (FDR) < 0.05 and fold-change in gene expression ≥ 2.

Supplementary Table S3: Diagnostic subgroup definition using gene expression-based score

Case	Histologic diagnosis	Score	Predicted diagnosis (threshold = 0.27)	Case	Histologic diagnosis	Score	Predicted diagnosis (threshold = 0.27)
18[V]	benign	0.00	benign	13[II,V]	PTC	0.56	PTC
19[T]	benign	0.00	benign	14[II,V]	PTC	0.49	PTC
20[V]	benign	0.09	benign	15[II,V]	PTC	0.51	PTC
21[T]	benign	0.13	benign	16[II,T]	PTC	0.57	PTC
22[T]	benign	0.10	benign	17[II,V]	PTC	0.67	PTC
23[V]	benign	0.17	benign	35[I,T]	PTC	0.10	*benign*
24[T]	benign	0.09	benign	37[I,V]	PTC	0.31	PTC
25[V]	benign	0.25	benign	39[I,T]	PTC	0.29	PTC
26[T]	benign	0.22	benign	41[I,T]	PTC	0.50	PTC
27[T]	benign	0.27	benign	43[I,V]	PTC	0.28	PTC
28[T]	benign	0.25	benign	45[II,V]	PTC	0.58	PTC
29[V]	benign	0.20	benign	47[II,V]	PTC	0.51	PTC
30[T]	benign	0.15	benign	49[II,V]	PTC	0.61	PTC
31[V]	benign	0.15	benign	51[II,V]	PTC	0.56	PTC
32[T]	benign	0.20	benign	53[II,T]	PTC	0.51	PTC
33[V]	benign	0.26	benign	55[II,V]	PTC	0.53	PTC
34[V]	benign	0.22	benign	57[II,T]	PTC	0.61	PTC
1[I,V]	PTC	0.61	PTC	59[I,V]	PTC	0.50	PTC
2[II,V]	PTC	0.51	PTC	61[I,T]	PTC	0.37	PTC
3[II,T]	PTC	0.95	PTC	63[I,V]	PTC	0.30	PTC
4[II,T]	PTC	0.63	PTC	65[I,T]	PTC	0.23	*benign*
5[II,T]	PTC	0.55	PTC	67[I,T]	PTC	0.38	PTC
6[II,V]	PTC	0.58	PTC	69[I,V]	PTC	0.14	*benign*
7[II,T]	PTC	0.71	PTC	71[II,T]	PTC	0.36	PTC
8[I,T]	PTC	0.63	PTC	73[II,V]	PTC	0.65	PTC
9[II,T]	PTC	0.63	PTC	75[II,T]	PTC	0.49	PTC
10[II,T]	PTC	0.72	PTC	77[II,V]	PTC	0.19	*benign*
11[II,V]	PTC	0.63	PTC	79[II,T]	PTC	0.47	PTC
12[II,T]	PTC	0.55	PTC	81[II,V]	PTC	0.46	PTC

[T]training set, [V]validation set, [I]less aggressive PTC, [II]more aggressive PTC
